# Development of a Current Injection—Type Impedance Measurement System for Monitoring Soil Water Content and Ion Concentration

**DOI:** 10.3390/s22093509

**Published:** 2022-05-05

**Authors:** Ryuki Shigemasu, Yuki Teraoka, Satoshi Ota, Harutoyo Hirano, Keita Yasutomi, Shoji Kawahito, Masato Futagawa

**Affiliations:** 1 Department of Engineering, Graduate School of Integrated Science and Technology, Shizuoka University, 3-5-1 Johoku, Naka-ku, Hamamatsu 432-8561, Japan; shigemasu.ryuki.16@shizuoka.ac.jp (R.S.); teraoka.yuki.16@shizuoka.ac.jp (Y.T.); 2 Department of Electrical and Electronic Engineering, Graduate School of Engineering, Shizuoka University, 3-5-1 Johoku, Naka-ku, Hamamatsu 432-8561, Japan; ota.s@shizuoka.ac.jp (S.O.); hirano.harutoyo@shizuoka.ac.jp (H.H.); yasutomi.keita@shizuoka.ac.jp (K.Y.); kawahito@idl.rie.shizuoka.ac.jp (S.K.); 3 Research Division, Graduate School of Science and Technology, Shizuoka University, 3-5-1 Johoku, Naka-ku, Hamamatsu 432-8561, Japan

**Keywords:** precision agriculture, soil sensor, water content, ion concentration, soil transient response characteristics

## Abstract

This study was conducted with the aim of developing a circuit system that enables the measurement of the moisture content and ion concentration with a simple circuit configuration. Our previous studies have shown that soil can be represented by an equivalent circuit of a parallel circuit of resistors and capacitors. We designed a circuit that can convert the voltage transient characteristics of the soil when a current is applied to it into a square wave and output frequency information and developed an algorithm to analyze the two types of square waves and calculate R and C. Normal operation was confirmed in the range of 10 kΩ–1 MΩ for the designed circuit, and the calculation algorithm matched within a maximum error of 5%, thus confirming the validity of the program. These successfully confirmed the changes in the water content and ionic concentration. The soil moisture content measurement succeeded in measuring a maximum error of about 10%, except at one point, and the soil ion concentration measurement succeeded in measuring a maximum error of 6.6%. A new, simple, noise-resistant moisture content and ion concentration measurement circuit system with square wave output has been realized.

## 1. Introduction

**Figure 1:**
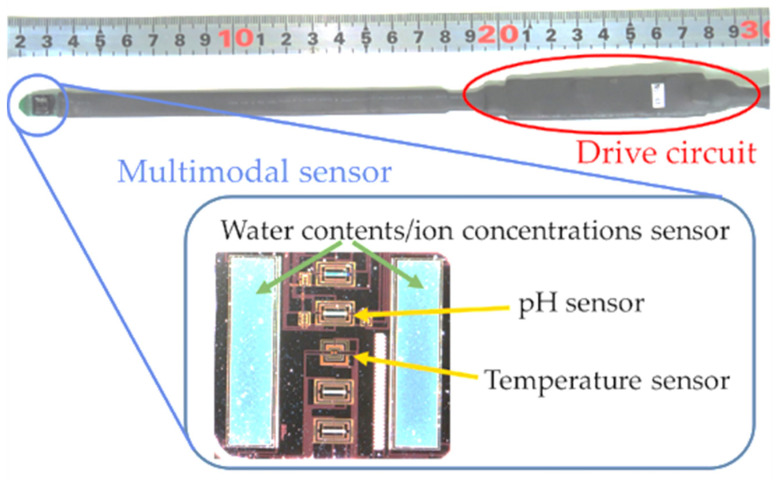
Multimodal sensor.

Precision agriculture is expected to make a significant contribution to improving the productivity and reducing the burden on farmers. In addition, precision agriculture requires sensing that is capable of wide-range and multipoint measurements. In particular, the use of the water content and ion concentration sensors to improve crop productivity has attracted considerable attention. In agriculture, the soil water content and ion concentration—that is, nutrient concentrations—have a significant impact on crop growth. Topp et al. and Munoz-Carpena studied the relative permittivity and conductivity of soils, and various soil sensors have been developed on the basis of their studies [1,2]. The time domain reflectometry (TDR) sensor is a typical sensor for the water content and ion concentration [3,4,5,6]. The sensor applies 1 MHz–1 GHz step pulses to the soil. It measures the water content based on the time it takes the pulse to travel through the soil. It also measures the conductivity, which changes with the water content and ion concentration changes based on the attenuation of the signal. The measurement system consists of an oscillator to generate step pulses, a sampling circuit to acquire data, and an oscilloscope making the device large. Another method is to use the capacitive sensor [7,8,9,10], which utilizes the capacitive nature of water-containing soil. Sensors that use capacitive touch-integrated circuits (ICs) [11,12] are cheaper and smaller than TDR sensors, but they are limited to measuring only the water content. The in situ measurements of the water content in soil by nuclear magnetic resonance has also been investigated [13,14,15]. To develop a compact sensor that can measure both the water content and ion concentration, our group focused on the impedance measurement of the soil. There are several methods of impedance measurement [16], such as amplitude domain reflectometry (ADR) [17], auto-equilibrium bridge, resonance, and IV measurements. The ADR method and the auto-equilibrium bridge method have the problem that the systems have become larger. The resonance method has the problem that it is limited to the measurement of the water content. These methods are not suitable for sensing, which is the goal of this research group. On the other hand, the IV method is based on a simple measurement principle and can be miniaturized. In addition, both the water content and ion concentration can be measured. Therefore, our research group has determined the water content and ion concentration from the impedance of soil using a commercially available impedance measurement IC [18] based on the IV method. By combining this method with a temperature sensor and a pH sensor, we have developed a multimodal sensor that can measure the temperature, water content, ion concentration, and pH in real time (Figure 1) [19,20,21,22]. The multimodal sensor uses Si-integrated circuit technology on a small 5-mmsquare substrate to integrate sensors for the simultaneous measurements of the temperature, water content, ion concentration, and pH. This sensor chip was used in our previous research to measure the water content of a tomato culture medium and to predict landslides [23]. However, there is a problem with the measurement of the water content and ion concentration using the impedance measurement IC described above. The impedance is measured by applying an AC voltage of 1 Hz–100 kHz, but the measurement range is limited, because an AC voltage of 1 MHz or higher is required for measurements in soil with a low water content or high ion concentration. In this study, we focused on the measurement of soil impedance using the voltage transient characteristics when a constant current is applied to the soil. We developed a simple and integrated measurement circuit that does not require a large oscillator and can handle frequencies of 1 MHz and above. We described the principle of the circuit operation and discussed the accuracy and the precision of the measurement results in this paper. The results showed that the proposed circuit and measurement system were able to monitor the water content and nutrient concentration in a soil medium with the aim of increasing the yield in agriculture.

## 2. Principle

### 2.1. Soil Impedance and Water Content/Ion Concentration

**Figure 2:**
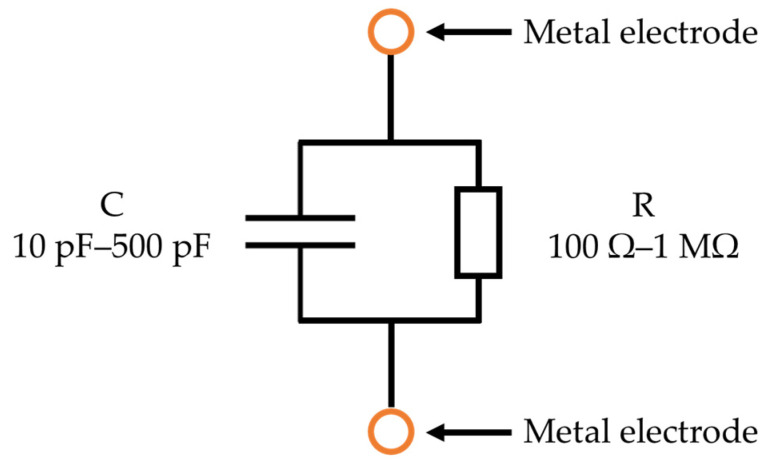
Equivalent circuit of the soil.

Since the relative permittivity of air is 1, that of water is 70–80, and that of soil is 3–7, and the water content of soil, which is the volume of water, can be determined from the combined capacity of the soil, water, and air [24]. The ion concentration indicates the sum of all types of ion concentrations contained in the water. In the case of water alone, it is often measured as the electrical conductivity, which is the reciprocal of the resistivity. In addition to the ion concentration, the electrical conductivity of soil is also affected by the water content. As the ion concentration or the water content increases, the electrical conductivity increases and the resistivity decreases. We aimed to measure the soil impedance to monitor the soil water content and the ion concentration. From our past research results, the electrical equivalent circuit of soil and water is represented as a parallel circuit of resistance R and capacitance C [23], from which the frequency characteristics are determined by the impedance measurement, as shown in Figure 2.

**Figure 3:**
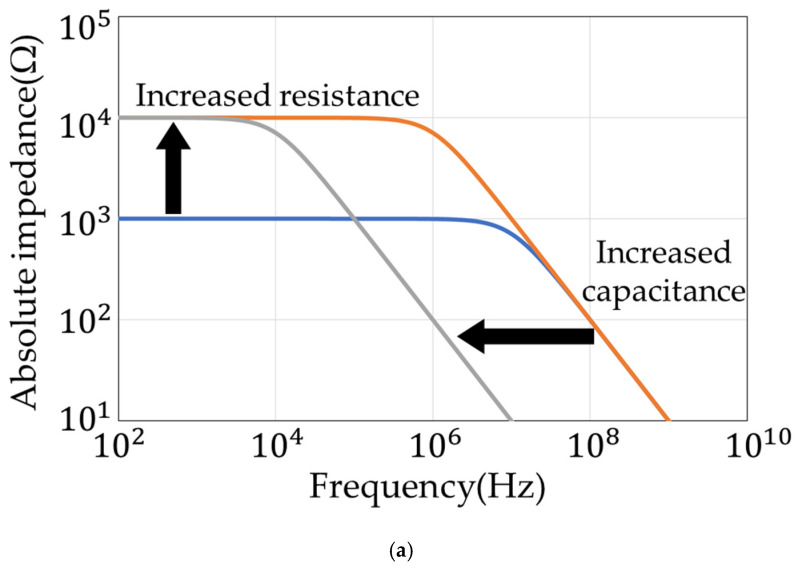

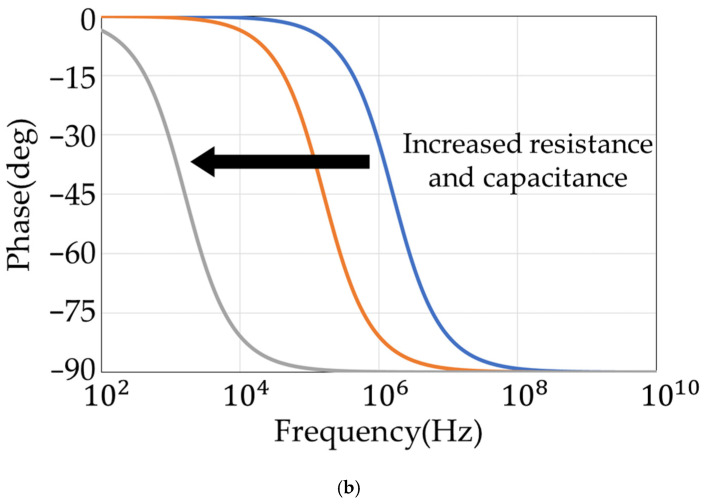
Frequency response of the R and C parallel circuit. (**a**) Absolute impedance. (**b**) Phase.

The resistance R has been found to be inversely proportional to the water content in the soil and to the ion concentration in the water. It varies in the range from 100 Ω to 1 MΩ. The capacitance C has been found to vary from 10 pF to 500 pF in proportion to the soil water content. It increases proportionally as the proportion of water increases. The absolute impedance and the phase of the frequency characteristics of the R and C parallel circuit are shown in Figure 3.

The value of R determines the impedance in the low-frequency region, and the value of C determines the impedance in the high-frequency region. To calculate R and C in this way requires impedance and phase measurements over a wide frequency range. In addition, measurements at high frequencies, such as above 10 MHz, are necessary, because the region of the C component moves to higher frequencies when the ion concentration is high.

### 2.2. Measurement Circuits

**Figure 4:**
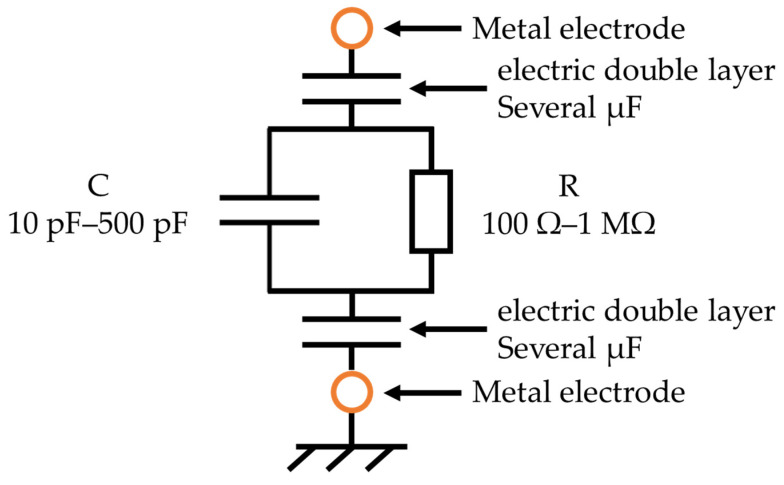
Equivalent circuit of the soil considering the electric double layer.

The R and C parallel circuit representing the impedance of soil and the capacitance of the electrical double layer generated at the sensor interface are represented by a series connection (Figure 4) [25].

**Figure 5:**
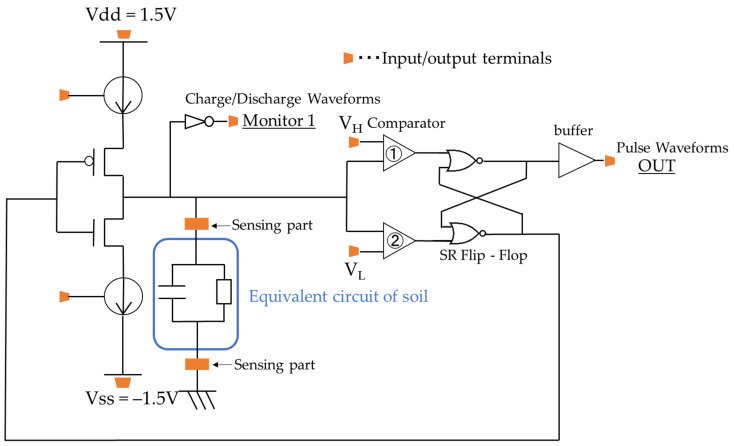
Measurement circuit.

We designed a circuit like the one shown in Figure 5, which allows us to measure the soil transient characteristics in a region unaffected by the electric double layer (above 10 kHz).

The CMOS switch in Figure 5 is designed using p-MOSFET and n-MOSFET, and when one is on, the other is off. When the p-MOSFET in Figure 5 is switched on, a positive current is applied to the soil from the current source. Then, the voltage of the soil rises and reaches the comparator’s threshold voltage VH. At this time, the comparator output is inverted and fed back into the switch. Then, the n-MOSFET in Figure 5 is switched on, and the voltage decreases, pulling the current out of the soil. Then, the negative threshold voltage VL of the comparator is reached, and the output is inverted. The circuit repeats the above behavior. As a result, the output of the comparator becomes a pulse waveform. Since the frequency changes depending on the changes in the R and C components of soil, we aim to determine the soil water content and ion concentration from the change in frequency. The electric double layer capacitance is on the order of µF, and the capacity of the soil is on the order of pF. Hence, the circuit is unaffected by the electric double layer capacitance, because the circuit is switched before the current is charged to the electric double layer capacitance. Comparators and buffers have a usable bandwidth of several tens of MHz. This makes it possible to measure soil with a low water content or high ion concentration that previously could not be measured with the commercially available impedance measurement ICs.

### 2.3. Soil Transient Characteristics When Current Is Applied

**Figure 6:**
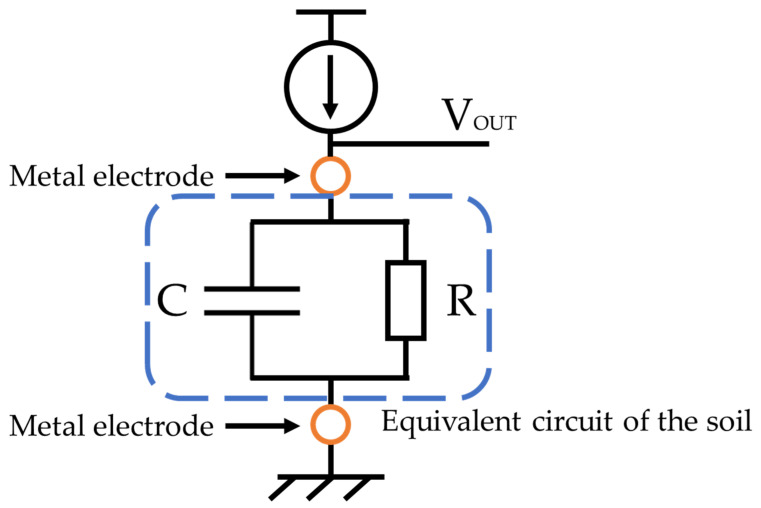
Equivalent circuit when a current is applied to the soil.

**Figure 7:**
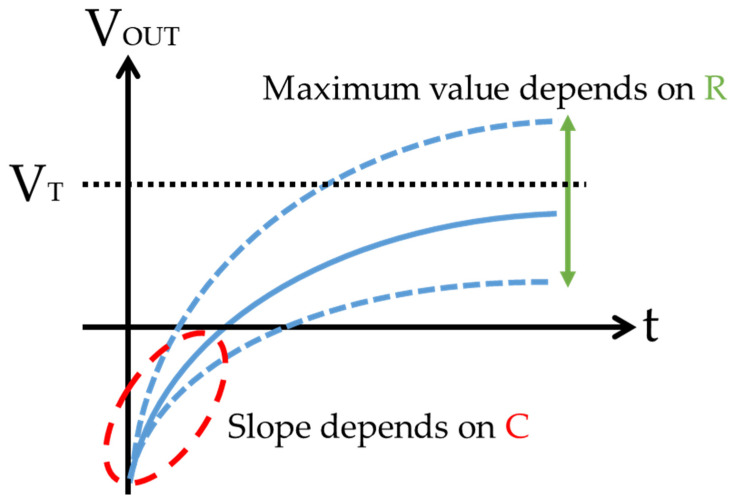
Voltage transient characteristics when the current is applied to the soil.

In Figure 6, when a constant current is applied when the initial voltage of V_OUT_ is negative, the voltage of V_OUT_ is increased. The transient characteristics of the V_OUT_ are shown in Figure 7. The slope of the transient characteristics of the voltage varies in the C component and is the maximum in the R component.

This voltage transient characteristic is expressed by

(1) $$V_{\mathrm{OUT}}=\left(\mathrm{RI}-V_{T}\right)\left(1-e^{-\frac{t}{\mathrm{RC}}}\right)-V_{T},$$

where R is the resistance of the soil, C is the capacitance of the soil, V_OUT_ is the output voltage (=V_T_), V_T_ is the threshold voltage, I is the applied current, and t is the time to reach the threshold voltage. Since there are two kinds of unknown R and C in Equation (1), we used two kinds of applied currents I and measured the time t taken to reach the threshold voltage V_T_.

## 3. Creation and Evaluation of the Circuit

### 3.1. Fabrication of Chips

**Figure 8:**
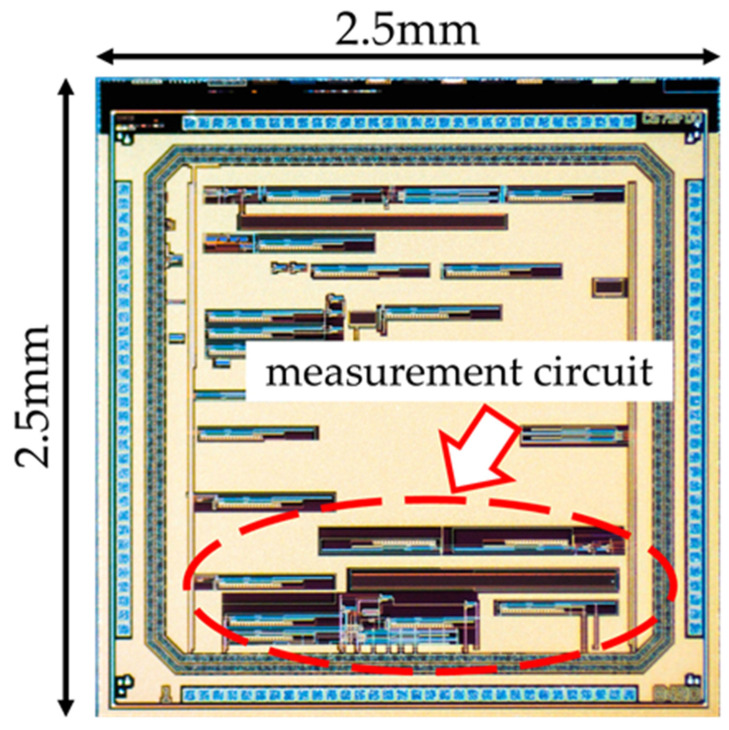
Designed chip (optical microscopy image).

We designed the layout to integrate the measurement circuit described in Section 2.2. The layout was designed using Cadence’s Virtuoso Layout Editor on the VDEC Rohm 0.18 µm process. The actual layout arrangement is shown in Figure 8. It is smaller in size than a sensor with a square chip of 2.5 mm per side. The measurement circuit is placed in the bottom part, and TEGs to verify elements such as the NOT, NAND, and NOR logic circuits and operational amplifiers are placed on the left side.

### 3.2. Checking the Operation of the Circuit

**Figure 9:**
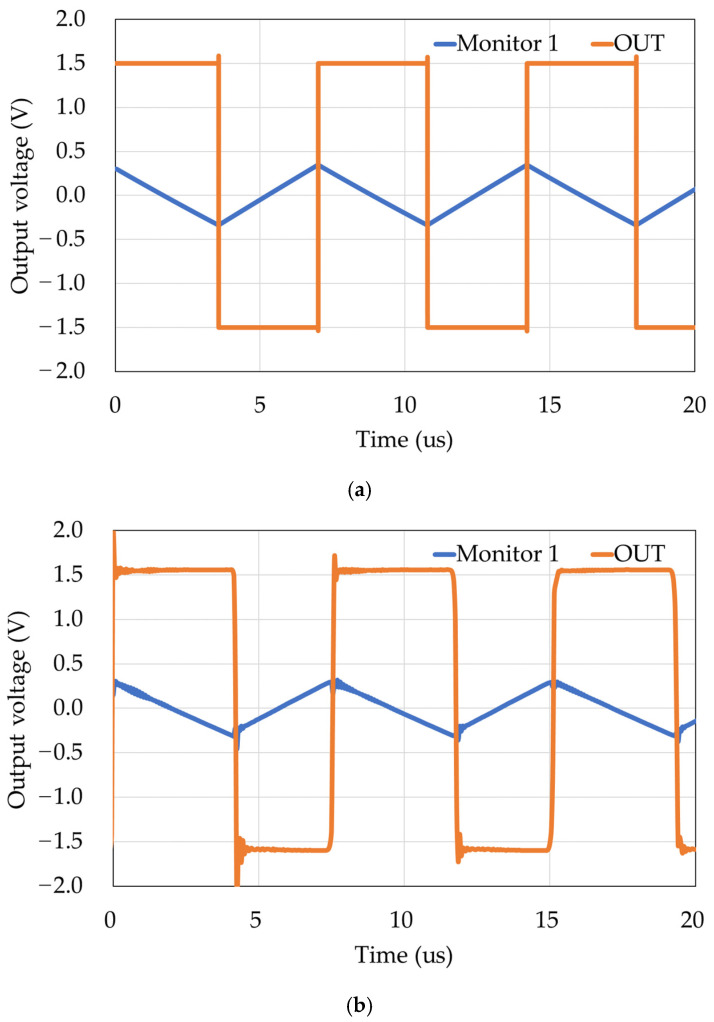
Output waveforms. (**a**) Simulation. (**b**) Actual measurements.

The operation of the finished chip was checked using resistors and capacitors that simulate the equivalent circuit of soil. Figure 9a shows the simulation results with R = 1 MΩ, C = 500 pF, applied current I = 100 μA, and threshold voltage V_T_ = 0.3 V. Figure 9b shows the actual measured results using discrete components under the same conditions. The output frequencies were 138.7 kHz for Figure 9a and 132.0 kHz for Figure 9b, and since the measured and simulated values were similar under the same conditions, we assumed that the fabricated chip was operating correctly. However, although the target measurement range for R is 100 Ω–1 MΩ, the range of our device is 10 kΩ–1 MΩ because we cannot measure below 1 kΩ owing to measurement circuit problems. In the future, the range will be improved to allow measurements using an amplifier.

### 3.3. Checking the Effect of Electric Double Layer Capacitance

**Figure 10:**
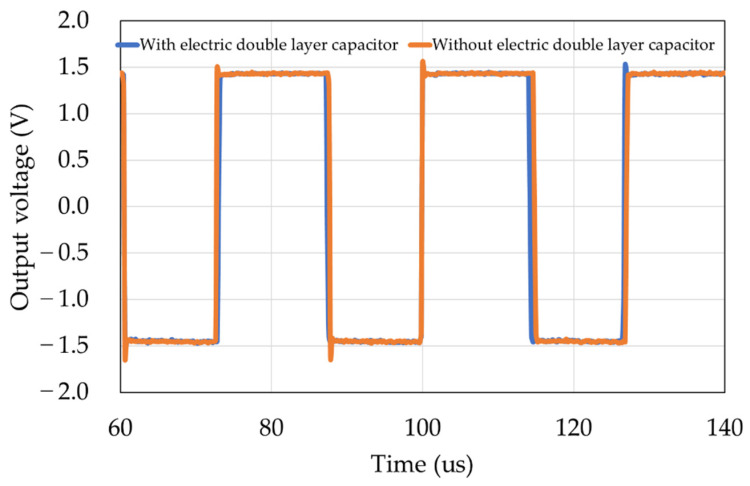
Results obtained with and without the electric double layer capacitor.

We conducted an experiment to confirm that the measurement circuit was not affected by the electric double layer described in Section 2.2. A capacitor (2.2 μF) was connected in a series to simulate the electric double layer using discrete components. Since the electric double layer tends to appear at lower frequencies, we changed R to 10 kΩ and I to 40 μA for the experiment described in Section 3.2. Figure 10 shows the results of superimposing the output waveforms of the measurement circuit. The measurement results showed good results, with an error of 1%.

## 4. RC Calculation Algorithm

We tried to solve Equation (1) in Section 2.3 mathematically, but it was difficult because two variables, R and C, were included in the equation. Thus, if we set V_T_ as V_OUT_ in Equation (1) and solve for C, we can transform it into Equation (2) to make it easier to calculate numerically.

(2) $$C=-\frac{t}{R\ln\left(1-\frac{2V_{T}}{\mathrm{RI}+V_{T}}\right)}.$$

**Figure 11:**
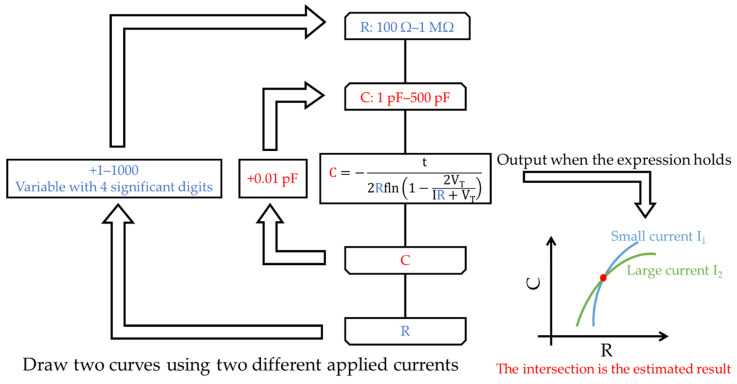
Flowchart of the RC calculation algorithm.

The two variables R and C were calculated numerically using Equation (2). Since the arrival time t is output as a frequency by the measurement circuit in Figure 5, we used the inverse of twice the frequency as the time t. The flowchart of the program for determining R and C from the applied current, threshold voltage, and output frequency of the measurement circuit is shown in Figure 11.

## 5. Simulation Experiments

**Figure 12:**
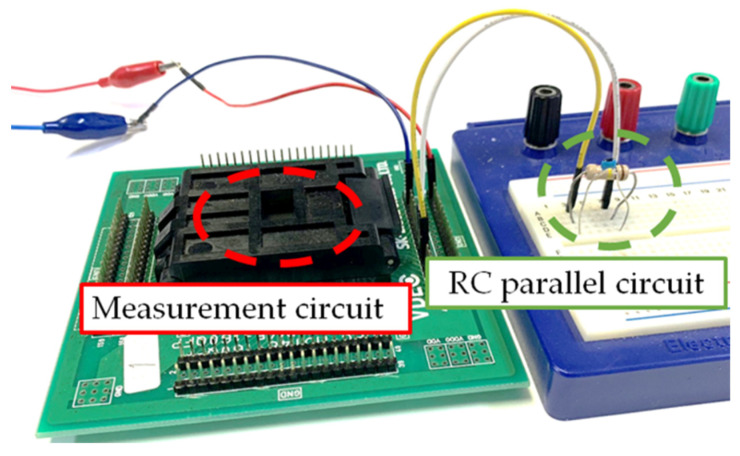
Measuring an RC parallel circuit using a measurement circuit.

**Figure 13:**
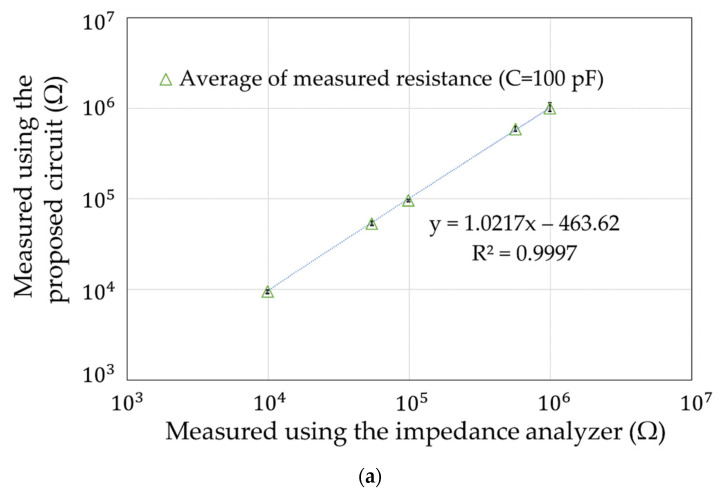

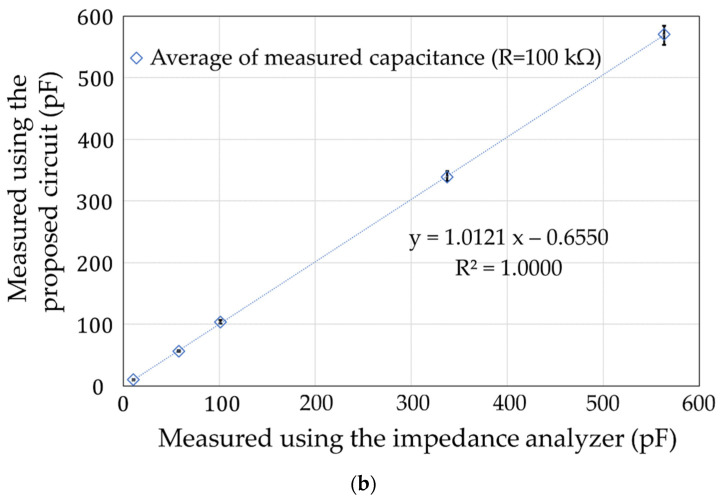
Calculation accuracy. (**a**) About resistance. (**b**) About capacitance.

**Figure 14:**
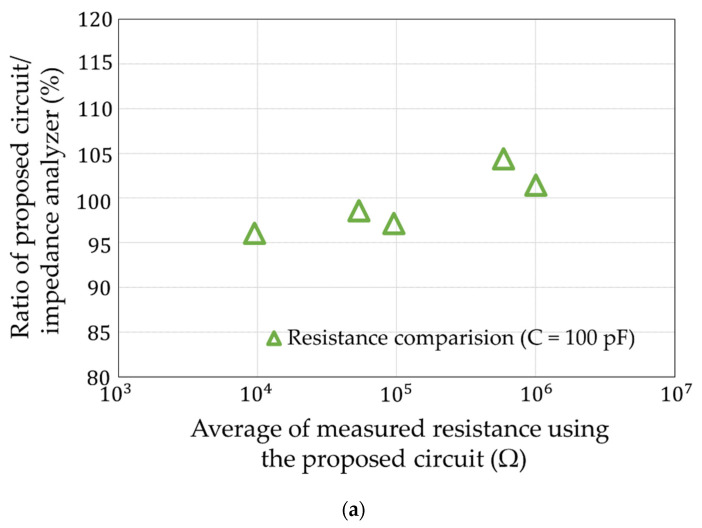

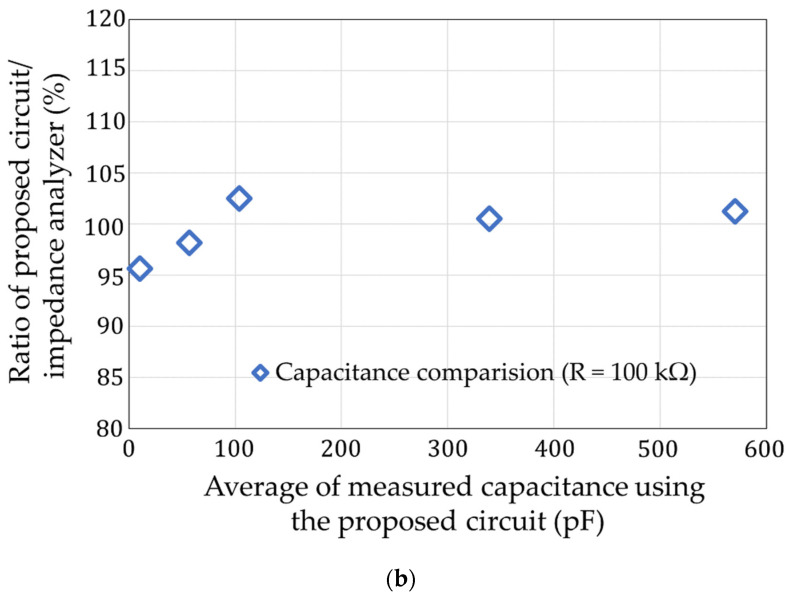
Deviation of the measurements with the impedance analyzer. (**a**) About resistance. (**b**) About capacitance.

**Figure 15:**
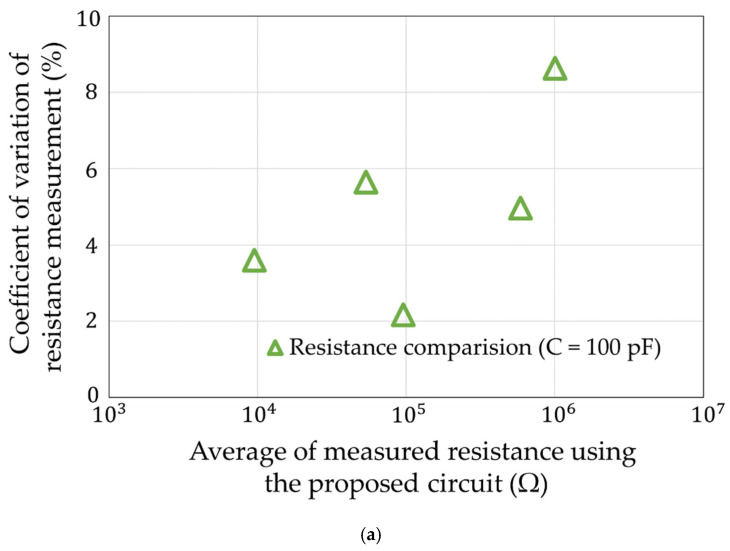

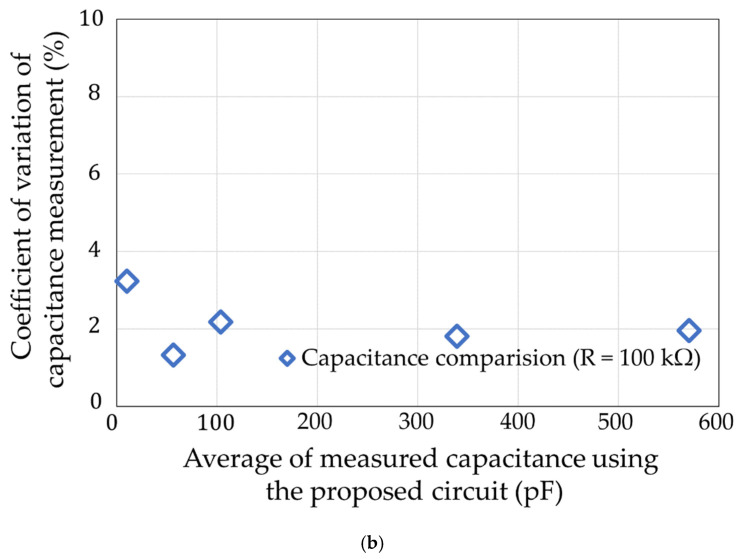
Coefficient of variation of Figure 14. (**a**) About resistance. (**b**) About capacitance.

To verify the proposed algorithm, an RC parallel circuit simulating the equivalent circuit of soil was fabricated and connected to the measurement circuit, as shown in Figure 12. The measurement range of the resistance and the capacitance has been found to vary from 100 Ω to 1 MΩ and from 10 pF to 500 pF from past experiments, so the measurements were made in these ranges. Using two types of applied currents, we measured the frequency and calculated the resistance and the capacitance values using the program. The results are shown in Figure 13, Figure 14 and Figure 15. Each measurement condition was measured five times. The maximum error was shown as an error bar in Figure 13. The ratio between the average measured value of our circuit and the measured value of the impedance analyzer is shown in Figure 14. The maximum error value was 95.6% at 10 pF and the 100 kΩ measurement condition. The coefficient of variation in each measurement condition is shown in Figure 15. The maximum error value was 8.6% at 100 pF and the 1 MΩ measurement condition. The validity of the accuracy of the program was confirmed by the fact that the values agreed within a maximum error of ±5% compared to the resistance and capacitance values measured with an impedance analyzer (Agilent Technologies 4294A). The proposed circuit could measure from 10 kΩ to 1.0 MΩ when 100 pF was used and from 11 to 507 pF when 100 kΩ was used.

## 6. Experiments to Measure Water Content and Ion Concentration

### 6.1. Measurement of Water Contents

**Figure 16:**
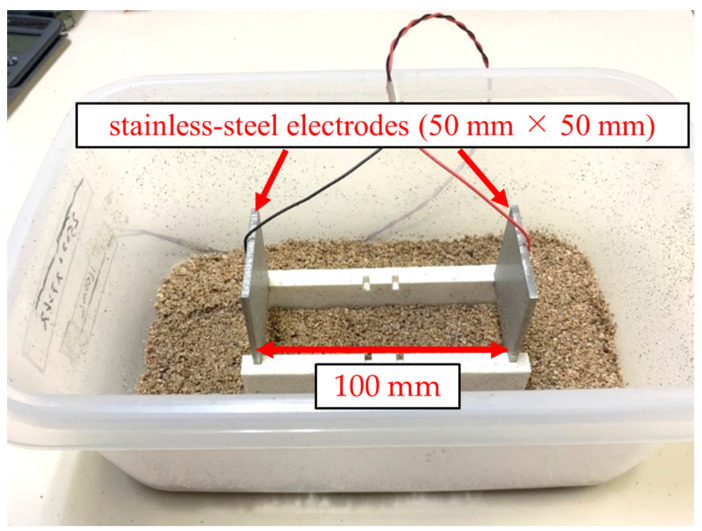
The experimental setup for the water content measurements.

To confirm the validity of the measurements of the water contents using the measurement circuit and RC calculation algorithm, a stainless-steel electrode (50 mm × 50 mm) was used to measure the moisture content in the soil. The water contents of the model soil were adjusted from 10% to 50% and embedded with stainless-steel electrodes at 100-mm intervals, as shown in Figure 16.

**Figure 17:**
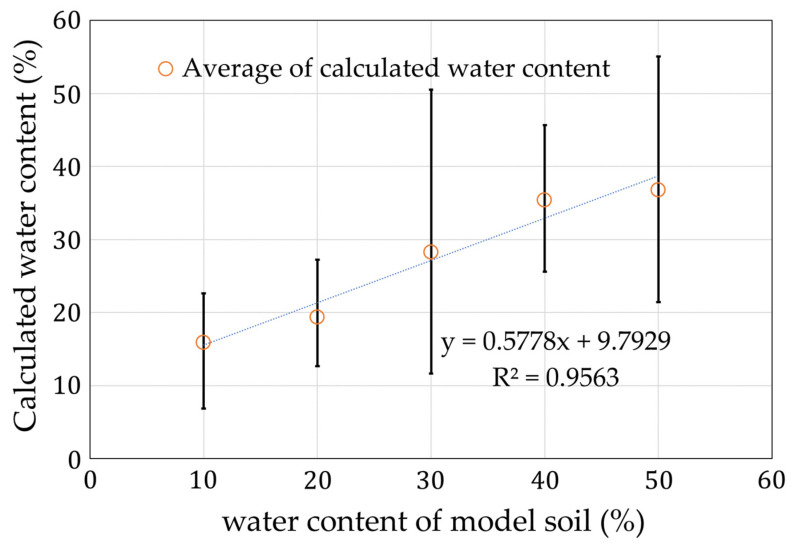
Results of the water content calculations.

Two types of applied currents were used to measure the frequency and calculate the resistance and the capacitance using the RC calculation program. The calculated capacitance was converted into a water content using the formula obtained in previous studies [7]. The results are shown in Figure 17. Each measurement condition was measured five times. The maximum error is shown as an error bar in the figure.

From the results in Figure 17, we successfully confirmed the increasing trend of the water content from measurements using the fabricated circuit and RC calculation program.

**Figure 18:**
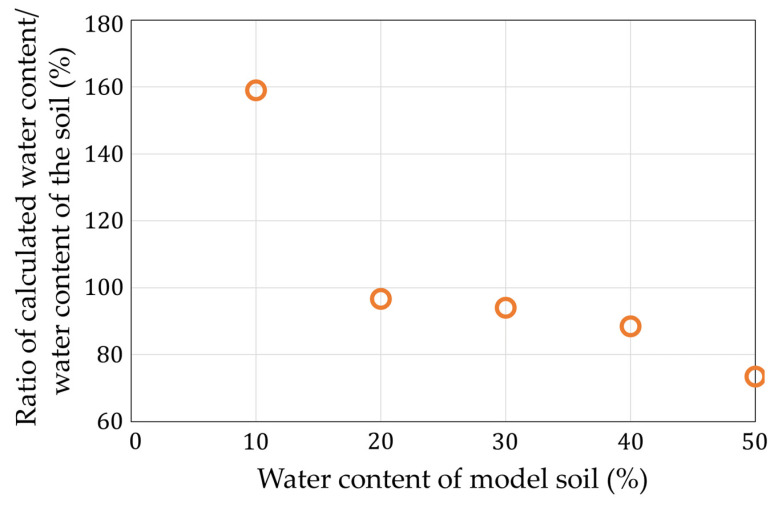
Deviation of the measurements with impedance analyzer in Figure 17.

**Figure 19:**
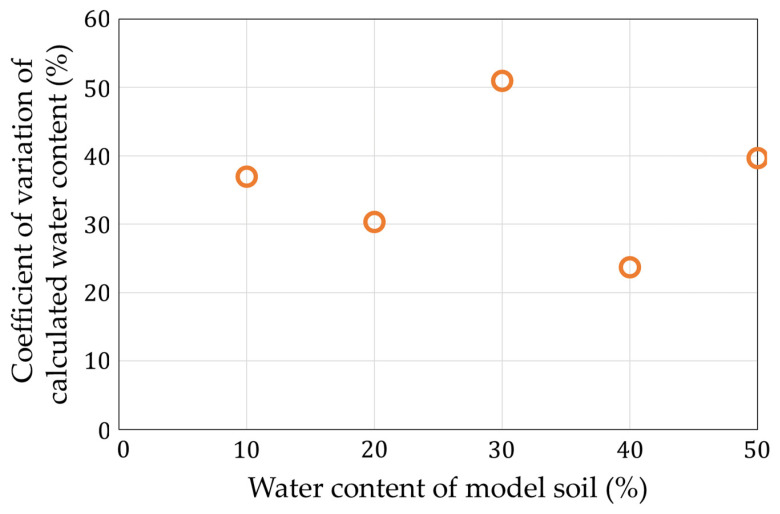
Coefficients of variation of Figure 17.

Figure 18 shows that the measurement of the lowest moisture content had a large error of 68%. At that point, the calculated capacitance value was 5.49 pF, which was the smallest among the calculated points. In addition, the measurement using the model soil required longer wiring, and the wiring capacitance was calibrated at the time of the calculation, but the calibration error may have affected the results. In the future, we would like to devise ways to reduce the influence of the wiring capacitance. Figure 19 shows the coefficient of variation of the water content. The maximum error value was 51% at a 30% water content. We estimated that the variations of all the measurement conditions became large because the noise went to the long wire.

### 6.2. Measurement of Ion Concentrations in Water

**Figure 20:**
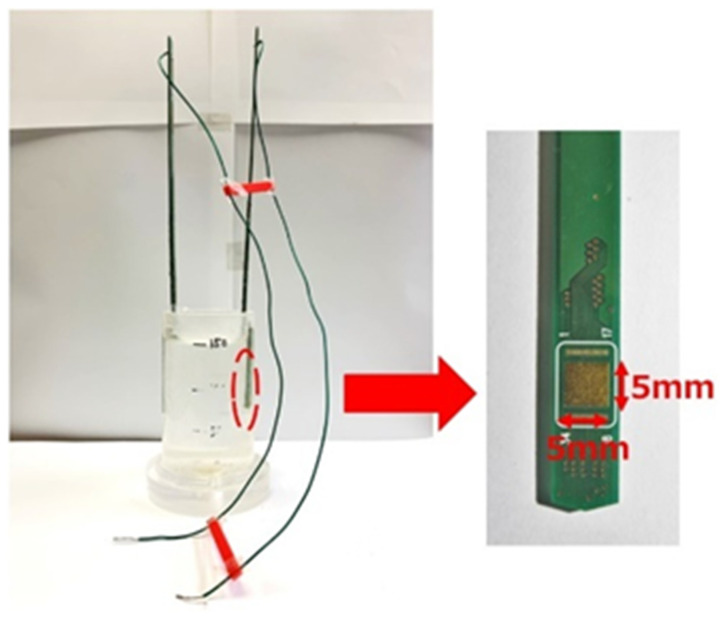
Measurement of the ion concentration in water using gold electrodes.

**Figure 21:**
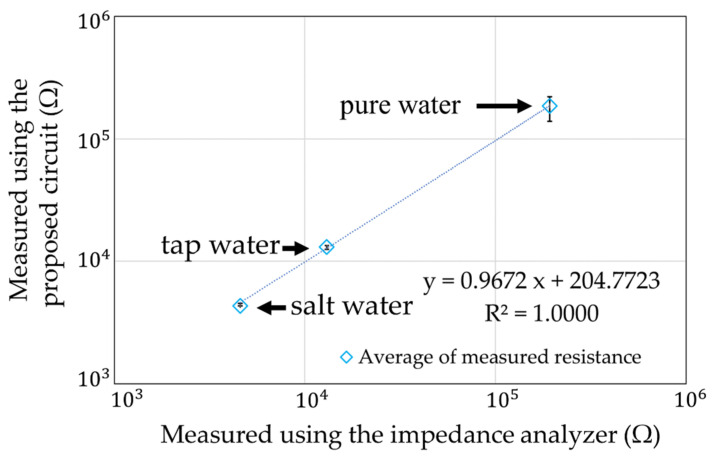
Measurement results of the ion concentration in water.

The ion concentrations in three samples of water were measured: pure water, tap water, and saltwater (40 mS/m). However, we used a gold electrode (5 mm × 5 mm), which was smaller than the stainless-steel electrode described in Section 6.1, to measure R, because the measurement circuit has a limited range of 10 kΩ–1 MΩ (Figure 20). This is because the voltage signal V_OUT_ in Figure 6 is small and does not reach the threshold in the range of 100–1 kΩ due to the limitation of the applied current. To enable measurements, future improvements are planned by using an amplifier. The results of the measurements using the measurement circuit and the impedance analyzer measurements are shown in Figure 21. Each measurement condition was measured five times. The maximum error is shown as an error bar in the figure. Experimentally, measurements could be made more than 4.5 kΩ. However, measurements could not be made in the range below that, because the threshold voltage was not reached due to the limitations of the applied current.

**Figure 22:**
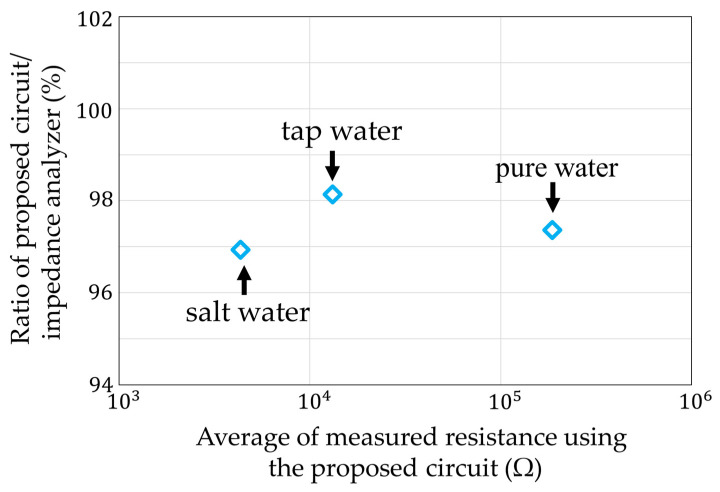
Deviation of measurements with impedance analyzer in Figure 21.

**Figure 23:**
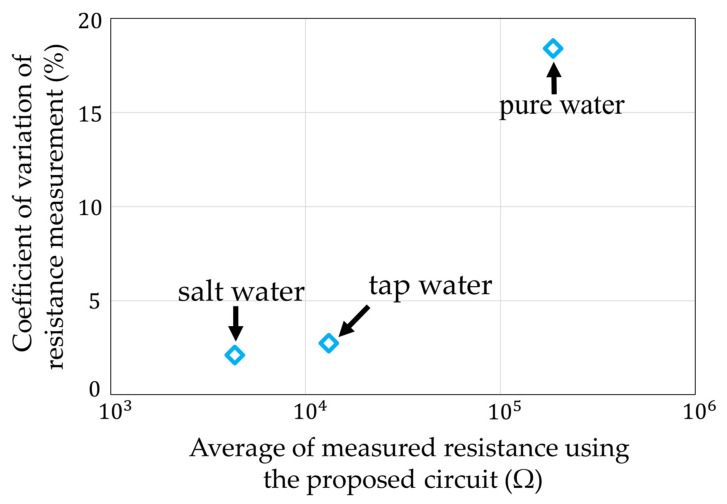
Coefficient of variation of Figure 21.

The ratio of between the average measured value of our circuit and the measured value of the impedance analyzer is shown in Figure 22. The maximum error value was 95.6% for the salt water. It was confirmed that the actual measurements using water were also possible. The coefficient of variation in each measurement condition is shown in Figure 23. The largest variation was 18.4% for the pure water. The other variations were less than 5%. It was estimated that the influence of noise mixed between the sensor electrodes was large, because the pure water had a high resistance.

### 6.3. Measurement Results of Ion Concentration in Soil

**Figure 24:**
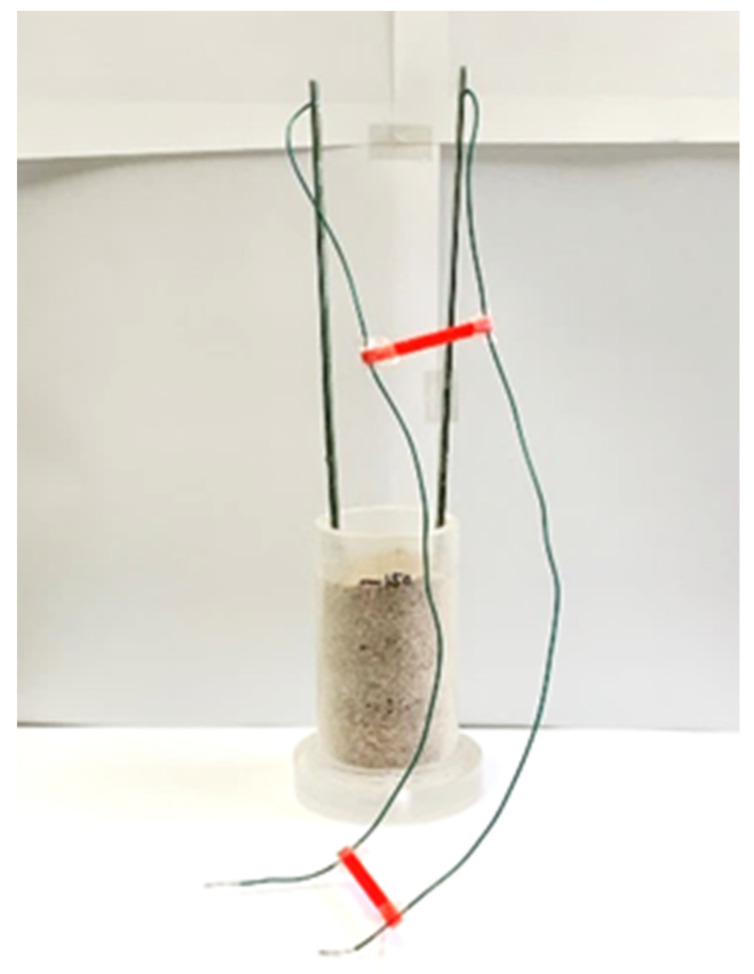
Measurement of the ion concentration in soil.

**Figure 25:**
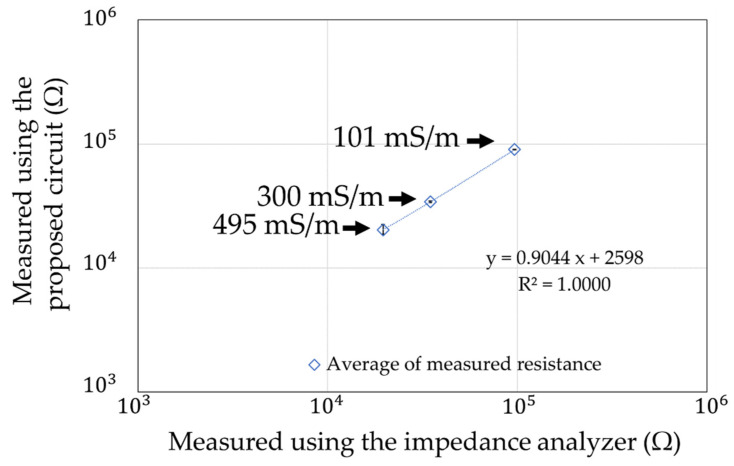
Measurement results of the ion concentration in soil.

Finally, we compared the resistance of soil containing water with different ion concentrations (100 mS/m, 300 mS/m, and 500 mS/m) (Figure 24). The gold electrode described in Section 6.2 was used for the measurements, with the soil water content set to 30%. The results, as well as the measurements with the impedance analyzer, are shown in Figure 25. Each measurement condition was measured three times. The maximum error is shown as an error bar in the figure.

**Figure 26:**
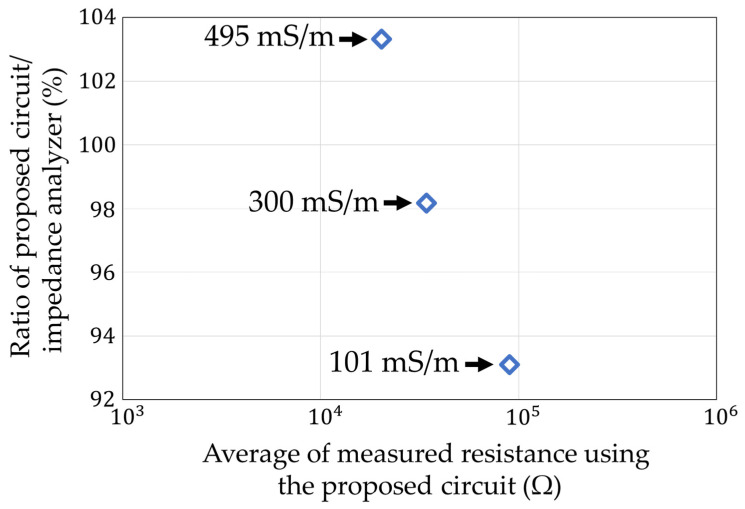
Deviation of the measurements with the impedance analyzer in Figure 25.

**Figure 27:**
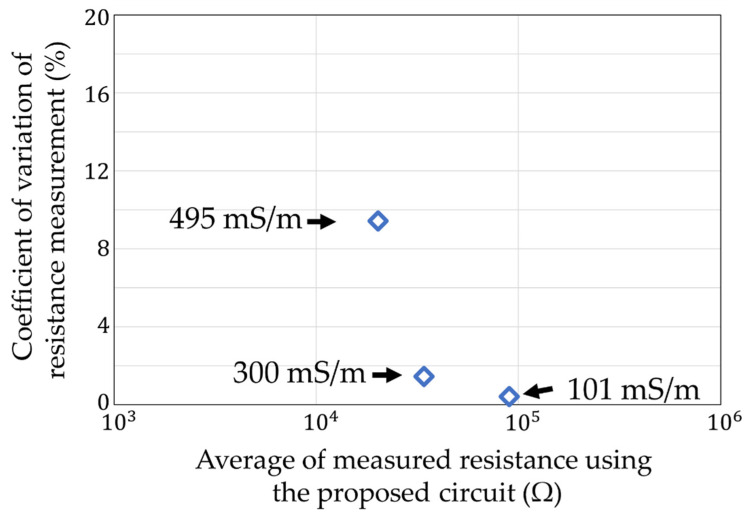
Coefficient of variation in Figure 25.

The ratio of between the average measured value of our circuit and the measured value of the impedance analyzer is shown in Figure 26. The maximum error value was 93.1% at 101 mS/m. The coefficient of variation in each measurement condition is shown in Figure 27. The largest variation was 9.4% at 495 mS/m. We could measure the ion concentration to measure the soil resistance below a 10% accuracy and precision.

## 7. Conclusions

A comparison with the other sensors is shown in Table 1.

Although the accuracy of the sensor is lower than those of other sensors, it does not require a high-frequency sine wave or an AD conversion circuit and is characterized by its simple system configuration and a design that is resistant to transmission noise. In addition, the simple structure and the feedback type made it possible to realize measurements that were unaffected by the electric double layer. Two types of output square waves were analyzed using the RC calculation algorithm to successfully capture changes in the water content and ion concentration. In the future, this measurement circuit, which is smaller than the sensors, is expected to enable the measurement of soil water contents and ion concentrations at multiple points deep underground, which has been difficult owing to transmission noise.

## Figures and Tables

**Table 1:** Comparison with the other sensors.

Measurement Method	Measurement Principle	Output	Noise Resistance	System Configuration	Accuracy
TDR	Transmission time	Reflected wave	○	Complex	±6%
AC voltage applied type	Impedance (absolute, phase)	Sine wave	×	Complex	±1.6%
DC current applied type (proposed circuit)	Voltage transient characteristics	Square wave	○	Simple	±5%

## Data Availability

Not applicable.
